# Bridging modalities: a deep learning framework for brain tumor classification via CT-MRI integration and model fusion

**DOI:** 10.3389/fncom.2026.1798561

**Published:** 2026-04-24

**Authors:** Ahmad Almadhor, Shtwai Alsubai, Najib Ben Aoun, Abdullah Al Hejaili, Amina Salhi, Tahani Alsubait, Fares Hamad Aljahani

**Affiliations:** 1 Department of Computer Engineering and Networks, College of Computer and Information Sciences, Jouf University, Sakaka, Saudi Arabia; 2 College of Computer Engineering and Sciences, Prince Sattam bin Abdulaziz University, AlKharj, Saudi Arabia; 3 Faculty of Computing and Information, Al-Baha University, Alaqiq, Saudi Arabia; 4 REGIM-Lab: Research Groups in Intelligent Machines, National School of Engineers of Sfax (ENIS), University of Sfax, Sfax, Tunisia; 5 Computer Science Department, Faculty of Computers & Information Technology, University of Tabuk, Tabuk, Saudi Arabia; 6 Department of Information Systems, College of Computer and Information Sciences, Princess Nourah bint Abdulrahman University, Riyadh, Saudi Arabia; 7 Department of Computer Science and Artificial Intelligence, College of Computing, Umm Al-Qura University, Makkah, Saudi Arabia; 8 Department of Information Systems, Faculty of Computing and Information Technology, Northern Border University, Rafha, Saudi Arabia

**Keywords:** brain tumor classification, convolutional neural network, CT-MRI integration, deep learning in neurology, model fusion, multimodal neuroimaging, ResNet18, transfer learning

## Abstract

Artificial intelligence (AI) and machine learning (ML) have shown remarkable promise in advancing medical image analysis, yet their potential in neurology and psychiatry remains underexplored. This work explores the use of deep learning approaches for automated brain tumor classification, leveraging multimodal neuroimaging data comprising computed tomography (CT) and magnetic resonance imaging (MRI) scans. Two model families were evaluated: a custom CNN trained from scratch and a transfer-learning approach based on ResNet-18. Models were trained and validated separately on CT and MRI datasets, and further extended to a combined dataset through multimodal fusion. Experimental results demonstrate that the CNN achieved accuracies of 97 and 99% on CT and MRI datasets, respectively, outperforming ResNet18, which yielded 95 and 97% under the same settings. On the combined dataset, CNN maintained superior performance (98%) compared to ResNet18 (94%), highlighting the adaptability of CNNs to domain-specific features in medical imaging. These findings suggest that lightweight CNNs can be highly effective for neuroimaging-based tumor detection, particularly when multimodal data are leveraged. Beyond clinical utility in early diagnosis, the authors underscore the importance of exploring modality-specific characteristics and model adaptability in designing AI-driven diagnostic systems for neurological disorders.

## 1 Introduction

Advances in artificial intelligence (AI) and machine learning (ML) are reshaping neuroimaging diagnostics by supporting automated, precise, and efficient identification of brain disorders (Rasool et al., 2024; Najib Ben Aoun et al., 2026; Krichen and Ben Aoun, 2026). Among these, brain tumor detection and classification remain areas of critical importance due to both the high morbidity and the clinical necessity of early and precise diagnosis. Imaging modalities such as computed tomography (CT) and magnetic resonance imaging (MRI) are standard in clinical workflows. Recent advances in deep learning, particularly convolutional neural networks (CNNs) and transfer learning architectures, have significantly improved classification accuracy. For example, hybrid models enhanced with explainable AI have achieved accuracies above 99% for multiclass tumor classification using MRI images (Gundogan, 2025; Rahman et al., 2025; Satushe et al., 2025; Rasool et al., 2025).

Despite these achievements, several critical gaps remain. First, performance on CT images often lags that on MRI due to lower contrast, different acquisition protocols, and modality-specific noise. Many studies focus solely on MRI, leaving CT comparatively underexplored in AI-driven tumor classification pipelines (Satushe et al., 2025; Kar and Singh, 2025; Aamir et al., 2025). Second, although pretrained models such as ResNet, EfficientNet, and Vision Transformers have shown strong results across multiple computer vision tasks, their effectiveness in neuroimaging is hindered by domain shift and dataset variability. Medical imaging data differ significantly from natural images, and features that are salient in one modality may not transfer well to another (Usha et al., 2024; Ibrahim et al., 2025). Furthermore, practical deployments face challenges such as limited dataset sizes, class imbalance, lack of standardized benchmarks, and concerns around interpretability, all of which hinder robust clinical adoption (Sankar et al., 2024).

To address these issues, multimodal integration leveraging both CT and MRI scans has gained increasing attention. Combining information from multiple imaging sources can improve generalization, mitigate modality-specific weaknesses, and provide complementary anatomical and pathological insights. However, multimodal fusion requires careful model design to account for differences in spatial resolution, intensity distributions, and acquisition artifacts across modalities. Recent investigations, such as MBTC-Net, demonstrate that multimodal classification can outperform single-modality approaches by integrating CT and MRI features with attention-based mechanisms (Kar and Singh, 2025; Usha et al., 2024). Similarly, CNN-TumorNet and I-BrainNet highlight the growing role of lightweight CNNs and IoT-enabled frameworks in advancing tumor classification with explainable AI, underscoring the clinical potential of interpretable deep learning systems (Rasool et al., 2025; Ibrahim et al., 2025).

In this work, we systematically investigate three experimental setups for brain tumor classification. First, we evaluate models trained independently on CT-only and MRI-only datasets, enabling benchmarking of modality-specific performance. Second, we explore training on a combined CT and MRI dataset using a custom CNN trained from scratch, thereby enabling us to assess the adaptability of lightweight architectures to heterogeneous inputs. Third, we employ a transfer-learning approach using a pretrained ResNet-18 model trained on the combined dataset and compare its performance with that of the CNN baseline. Finally, we evaluate a multimodel fusion scheme that aggregates predictions from both CNN and ResNet, leveraging their complementary strengths. Our results reveal that CNN models trained from scratch can outperform ResNet-18 in this setting, particularly when trained on the combined dataset, achieving approximately 99% accuracy. These findings contribute to the growing body of evidence that modality-aware and architecture-aware design choices are crucial for effective clinical AI systems in neurology and oncology. By highlighting the comparative strengths of CT, MRI, and multimodal integration, this study provides actionable insights for future AI-driven diagnostic frameworks and their potential integration into clinical practice.

### 1.1 Motivation

Brain tumor diagnosis remains a critical challenge in clinical practice, where timely and accurate classification of tumors can significantly influence patient outcomes. Although deep learning has achieved remarkable progress in medical imaging, most studies focus primarily on MRI due to its superior soft-tissue contrast, often neglecting CT despite its accessibility and clinical relevance. This modality imbalance, combined with reliance on pretrained networks such as ResNet, which were originally designed for natural images, introduces limitations related to domain shift, heterogeneous imaging protocols, and reduced generalization. Furthermore, while multimodal integration of CT and MRI holds promise for leveraging complementary information, existing approaches are limited by dataset imbalance, fusion complexity, and a lack of systematic comparisons between modality-specific and multimodal pipelines. These gaps motivate the present study, which benchmarks CNN and ResNet-18 on CT-only and MRI-only datasets, extends evaluation to a combined multimodal dataset, and explores multimodal fusion strategies to design robust, modality-aware, and clinically viable AI frameworks for brain tumor classification.

### 1.2 Contributions

The major contributions of this work are as follows:

- **Modality-specific benchmarking:** we provide a detailed evaluation of deep learning models trained separately on CT-only and MRI-only datasets, demonstrating that MRI yields higher classification accuracy due to its superior structural contrast, while CT remains an important complementary modality.
- **Custom CNN for domain adaptability:** a lightweight convolutional neural network (CNN) trained from scratch is developed, showing superior adaptability to medical imaging data compared to transfer learning with ResNet18. The CNN achieved up to 99% accuracy on MRI data, outperforming ResNet18 on the same dataset.
- **Multimodal sataset integration:** we construct a combined dataset of CT and MRI scans and systematically analyze performance when training on heterogeneous data. This highlights the benefits and challenges of modality integration for tumor classification.
- **Comparative transfer learning analysis:** by applying ResNet18 to both single-modality and multimodal datasets, we evaluate the effectiveness and limitations of pretrained models in handling domain shifts between natural images and medical scans.
- **Multimodel fusion strategy:** we explore a late-fusion approach that combines predictions from CNN and ResNet18 models. This scheme leverages complementary strengths of both architectures, improving robustness and highlighting ensemble learning as a viable path for clinical AI systems.

## 2 Related work

Recent advances in deep learning have significantly improved brain tumor classification and detection, particularly with MRI, which offers superior tissue contrast. Authors in Lu et al. (2025) proposed a deep learning-driven pipeline for MRI-based tumor classification and segmentation using non-contrast MRI, achieving high accuracy in both tasks. Authors in Rasa et al. (2024) fine-tuned transfer learning models on MRI datasets and reported strong performance across multiple tumor classes. The research in Albalawi et al. (2024) further proposed a customized multi-layer CNN architecture that improved classification accuracy while maintaining computational efficiency. Authors in Balamurugan et al. (2024) combined ResNet features with channel-wise attention to develop a robust classification model, achieving accuracy levels above 99%. These studies highlight the maturity of MRI-based pipelines and their consistent ability to reach near-perfect accuracy in controlled settings.

Explainable AI (XAI) has emerged as an important focus to improve transparency and clinical trust in medical imaging models. Authors in Akgündoğdu and Çelikbaş (2025) integrated Grad-CAM, LIME, and SHAP into an explainable MRI tumor-detection framework, thereby enhancing model interpretability. Authors in Iftikhar et al. (2025) developed an explainable CNN that identified key imaging features contributing to classification. In contrast, work in Majeed et al. (2025) introduced spatial-channel attention with transformer context to strengthen both performance and interpretability. Authors in Filvantorkaman et al. (2025) proposed a fusion-based deep learning system that combines explainable AI and rule-based reasoning, achieving improved accuracy while aligning with clinical decision processes. Beyond brain tumors, a study in Sritharan et al. (2025) demonstrated the effectiveness of XAI-driven segmentation for cervical cancer screening, reinforcing the broader value of interpretability across medical imaging tasks.

Hybrid and transfer learning approaches have also gained traction in improving the robustness of tumor classification. Authors in Khan et al. (2025) employed transfer learning for brain tumor classification from MRI scans, achieving improved diagnostic accuracy over conventional CNNs. Authors in Arora and Mishra (2025) developed a hybrid deep learning framework with self-adaptive noise resilience, highlighting the importance of robust architectures for real-world clinical imaging. Authors in Sun et al. (2024) introduced MVSI-Net, which uses multi-view attention and multi-scale feature interaction for tumor segmentation, demonstrating the benefits of leveraging contextual information across MRI views. Authors in Altin Karagoz et al. (2024) proposed ResViT, a residual vision transformer trained via self-supervised learning, demonstrating robustness and transferability across diverse MRI datasets. Authors in Tandel et al. (2025) applied majority-vote ensemble strategies for multiclass brain tumor grading, validated them using explainability, and demonstrated strong reliability across grade-classification tasks.

Real-time detection and deployment-oriented pipelines are becoming increasingly important. Authors in Cepeda et al. (2025) developed an intraoperative ultrasound detection framework optimized for real-time tumor detection in the operating room, directly addressing surgical needs. Authors Alnageeb and Supriya (2025) proposed a lightweight deep learning model tailored for real-time tumor classification, achieving high speed without sacrificing diagnostic accuracy. Similarly, Authors in Almufareh et al. (2024) applied YOLO-based deep learning for automated tumor segmentation and classification in MRI. In contrast, Authors in Ali and Zhang (2024) provided a comprehensive review of the YOLO family, highlighting its suitability for high-speed medical imaging tasks. These works emphasize the critical need to balance accuracy and computational efficiency for clinical integration.

Finally, surveys and systematic reviews provide a broader context. Authors in Bacon et al. (2024) analyzed AI-driven neuroimaging approaches, emphasizing challenges such as data variability, lack of standardization, and interpretability. Authors in Sailunaz et al. (2024) reviewed brain tumor image analysis, summarized state-of-the-art pipelines, and identified gaps in CT-based research. Collectively, these studies illustrate rapid advances in brain tumor classification but also highlight unresolved challenges: underutilization of CT in AI pipelines, limited exploration of multimodal integration, and the need for explainable, real-time solutions. These open challenges directly motivate our study, which benchmarks CNN and ResNet-18 on CT, MRI, and combined datasets and investigates multimodal fusion for robust, clinically relevant brain tumor classification.

## 3 Proposed methodology

**Figure 1:**
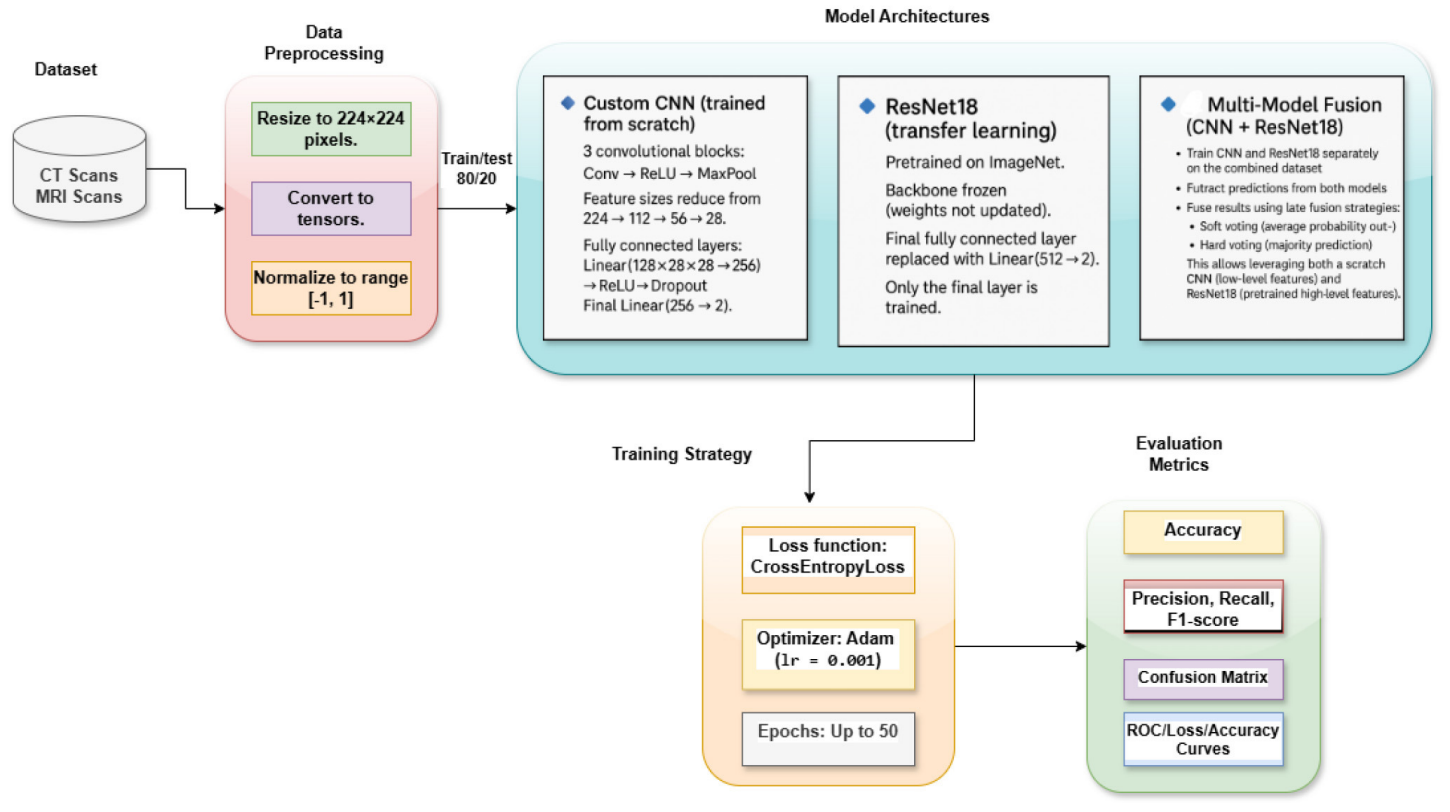
Proposed multimodal deep learning framework for brain tumor classification. The pipeline integrates preprocessing (resizing, tensor conversion, normalization), model architectures (CNN, ResNet18, and multimodel fusion), a standardized training strategy, and multi-metric evaluation.

Figure 1 presents the workflow of the proposed multimodal deep learning framework for brain tumor classification using CT and MRI scans. The pipeline begins with dataset sourcing and curation from publicly available repositories, where CT and MRI images are collected, standardized, and organized into tumor and healthy classes. All images are resized to 224 × 224 pixels, converted into tensors, and normalized to the range [−1, 1]. The dataset was split into training and test sets at an 80:20 ratio to ensure balanced model evaluation. Three model architectures are employed: (i) a custom CNN trained from scratch with three convolutional blocks (Conv → ReLU → MaxPool) followed by fully connected layers for binary classification; (ii) a ResNet18 model using transfer learning, where the ImageNet-pretrained backbone is frozen and only the final fully connected layer is replaced and fine-tuned; and (iii) a multimodel fusion approach that combines the predictions of CNN and ResNet18 using soft voting (average probability outputs) and hard voting (majority prediction), thereby exploiting complementary low-level and high-level features. Training is conducted with CrossEntropyLoss as the objective function and the Adam optimizer (*lr* = 0.001) for up to 50 epochs, with early stopping to prevent overfitting. Model performance is comprehensively evaluated using accuracy, precision, recall, and F1-score, while confusion matrices are generated to visualize class-specific performance. In addition, ROC curves and AUC values are computed to assess discriminative ability under varying thresholds.

Algorithm 1 outlines the complete workflow of the proposed multimodal deep learning framework for brain tumor classification using CT and MRI scans. The process begins with dataset initialization, during which CT and MRI images are collected, organized into tumor and healthy categories, and stored in a directory structure compatible with PyTorch ImageFolder. Following preprocessing, three model configurations are prepared. The first is a custom CNN trained from scratch, comprising three convolutional blocks (Conv → ReLU → MaxPool) followed by fully connected layers that directly learn discriminative features from CT and MRI data. The second is a ResNet-18 model based on transfer learning, in which the ImageNet-pretrained backbone is frozen, and the final fully connected layer is replaced with a binary classifier, enabling the model to adapt pretrained high-level features to medical imaging. We further implement a CNN-ResNet18 fusion model that combines independently trained networks using ensemble decision strategies, namely soft and hard voting. This fusion exploits complementary feature representations, low-level spatial features from CNN, and high-level semantic features from ResNet18. The models are trained using CrossEntropyLoss as the objective function and optimized with Adam (*lr* = 0.001) for up to 50 epochs. Early stopping is employed to terminate training when validation loss stagnates across epochs, thereby preventing overfitting and promoting efficient convergence. Once trained, model performance is evaluated using a comprehensive set of metrics, including accuracy, precision, recall, and F1-score. Confusion matrices are used to analyze class-wise prediction performance, whereas ROC curves and AUC values provide insight into discriminative capability across different decision thresholds. The fusion model is validated against individual CNN and ResNet18 baselines to confirm performance gains.

Algorithm 1 **Multimodal brain tumor classification using CNN, ResNet18, and fusion.**

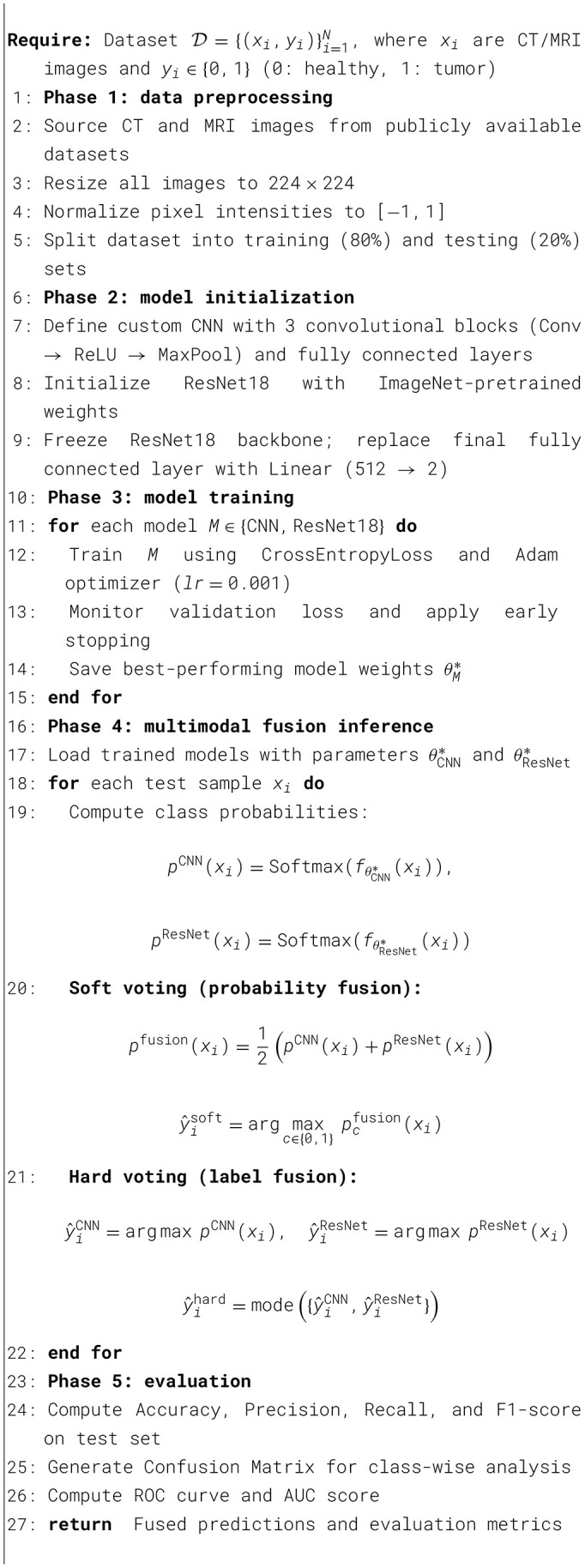

### 3.1 Data collection and preprocessing

The proposed study uses a curated multimodal dataset of brain tumor images from publicly available repositories, primarily the dataset from Murtoza (2020). No new clinical data were collected in this study. Instead, CT and MRI images were sourced, aggregated, and curated from existing open-access datasets to ensure diversity and coverage across imaging modalities. The dataset consists of two modalities. The CT subset contains 4,618 images, including 2,300 healthy and 2,318 tumor samples, with a nearly balanced distribution. The MRI subset comprises 5,000 images, including 2,000 healthy and 3,000 tumor samples, indicating a moderate class imbalance toward tumor cases. Overall, the combined multimodal dataset contains 9,618 images. While the CT dataset is well-balanced, the MRI dataset reflects a slightly imbalanced yet realistic clinical distribution. Each image is labeled as either *tumor* or *healthy*, enabling binary classification across modalities. All images are preprocessed by resizing to a uniform resolution of 224 × 224 pixels and converted into tensors for model compatibility. Intensity values are normalized to the range [−1, 1], ensuring stable training and consistent feature scaling across modalities. The dataset is partitioned into training and test sets using an 80:20 split to ensure consistent evaluation across all experimental settings, including CT-only, MRI-only, and multimodal configurations. This fixed split allows fair comparison between models while maintaining computational efficiency. The overall preprocessing pipeline ensures consistency across CT and MRI modalities, mitigates scale variance, and provides a robust foundation for multimodal deep learning experiments.

### 3.2 Proposed multimodal classification model

The proposed brain tumor classification framework integrates two complementary deep learning architectures: a custom Convolutional Neural Network (CNN) trained from scratch and a transfer-learning model based on ResNet-18. Given an input image *I*∈ℝ^224 × 224 × 3^, the goal of the model is to predict the class label *y*∈{0, 1}, where 0 represents *healthy* and 1 represents *tumor*. Formally, the model learns a mapping function defined as in Equation 1:


$$\begin{array}{l} f_{\theta }:I\to y \end{array}$$ (1)

where θ denotes the trainable parameters of the network.

The custom CNN architecture is intentionally designed to be lightweight and domain-adaptive, enabling it to learn modality-specific features directly from medical images without relying on pretrained representations. Unlike standard transfer learning models, which may suffer from domain shift due to differences between natural and medical images, the CNN focuses on capturing fine-grained spatial and intensity patterns specific to CT and MRI scans. It consists of three convolutional blocks of the form Conv → ReLU → MaxPool, progressively reducing spatial dimensions while increasing channel depth. The extracted feature maps are flattened and processed by fully connected layers with dropout regularization before the final classification stage. This design provides sufficient representational capacity while maintaining computational efficiency and reducing the risk of overfitting, particularly for heterogeneous medical datasets.

The ResNet18 architecture is selected as a representative transfer-learning baseline due to its strong balance of performance, computational efficiency, and architectural simplicity. Compared to deeper variants such as ResNet-50 or DenseNet, ResNet-18 has fewer parameters, reducing the risk of overfitting while still benefiting from residual connections that enable effective feature extraction. Its convolutional backbone, pretrained on ImageNet, is frozen to retain generic visual representations, and the final fully connected layer is replaced with a binary classifier in Equation 2:


$$\begin{array}{l} z=W\cdot h+b \end{array}$$ (2)

where (h) is the 512-dimensional feature vector from the global average pooling layer, (W) and (b) are learnable parameters, and (z) represents the logits for the two output classes. This setup allows the model to adapt pretrained high-level features to medical imaging while maintaining training stability and efficiency.

The training objective for both CNN and ResNet18 models is the binary cross-entropy loss in Equation 3:


$$\begin{array}{l} ℒ=-\frac{1}{N}\sum_{i=1}^{N}\left[y_{i}\log\left({\hat{y}}_{i}\right)+\left(1-y_{i}\right)\log\left(1-{\hat{y}}_{i}\right)\right] \end{array}$$ (3)

where (*y*_*i*_) is the ground-truth label and (ŷ_*i*_) is the predicted probability for sample (*i*).

To exploit complementary feature representations, a multimodel fusion strategy is introduced. Predictions from CNN (*p*^*CNN*^) and ResNet18 (*p*^*ResNet*^) are aggregated using late fusion. In **soft voting**, the probabilities are averaged in Equation 4:


$$\begin{array}{l} \hat{y}=\arg\max\left(\frac{1}{2}\left(p^{CNN}+p^{ResNet}\right)\right) \end{array}$$ (4)

while in **hard voting**, the final decision is made by majority rule between the two classifiers. Although similar peak accuracy is observed for MRI-only CNN and multimodal fusion, the fusion framework operates on a more heterogeneous CT–MRI dataset and benefits from combining domain-specific features from CNN with robust pretrained representations from ResNet18. This results in improved generalization, stability, and robustness across modalities, making the system more suitable for real-world clinical scenarios where imaging conditions may vary.

To improve generalization, data augmentation techniques, including random flips, rotations, and intensity normalization, are applied during training. Early stopping is employed to prevent overfitting, and the best-performing checkpoints are retained. Performance is evaluated using Accuracy, Precision, Recall, F1-score, and ROC-AUC to ensure a robust and clinically relevant assessment of classification performance across CT-only, MRI-only, and combined datasets.

## 4 Experimental analysis, and results

The results in Table 1 demonstrate clear performance differences across models and imaging modalities. For CT scans, the CNN achieved an overall accuracy of 94%, with balanced precision, recall, and F1-scores of 0.94, indicating reliable but slightly lower sensitivity for tumor cases. In comparison, ResNet18 on CT achieved modest improvements, with 95% accuracy and macro-averaged precision, recall, and F1 Scores of 0.95, benefiting from its pretrained backbone. MRI-based models showed substantially better performance: CNN attained 98% accuracy with precision = 0.98, recall = 0.97, and F1-score = 0.97, while ResNet18 reached 97% accuracy with equally balanced metrics of 0.97 across precision, recall, and F1-score. The multimodel fusion of CNN and ResNet18, trained on the combined CT-MRI dataset, delivered the best results, achieving 99% accuracy, with precision, recall, and F1-scores all at 0.99. These findings highlight three key insights: (i) MRI consistently outperforms CT due to superior soft-tissue contrast, (ii) CNN trained from scratch adapts better to domain-specific tumor features compared to transfer learning with ResNet18, and (iii) fusion of CNN and ResNet18 leverages complementary feature representations to deliver state-of-the-art performance, minimizing both false negatives and false positives in brain tumor classification.

**Table 1:** Comparison of classification performance across CNN, ResNet18, and Fusion models on combined datasets.

Model/dataset	Accuracy	Precision	Recall	F1-score
CNN (CT)	0.94	0.94	0.94	0.94
CNN (MRI)	0.98	0.98	0.97	0.97
ResNet18 (CT)	0.95	0.95	0.95	0.95
ResNet18 (MRI)	0.97	0.97	0.97	0.97
Fusion multimodel(CNN + ResNet)	**0.99**	**0.99**	**0.99**	**0.99**

The best results are shown in bold.

**Figure 2:**
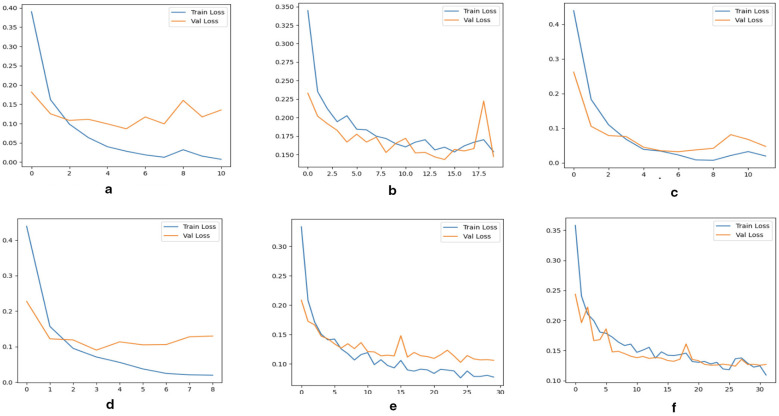
Training vs. validation loss curves for CNN, ResNet18, and multimodel fusion across combined datasets. The figures illustrate consistent convergence and generalization behavior across all experiments. **(a)** Multimodel CNN loss, **(b)** multimodel ResNet18 loss, **(c)** CNN (MRI) loss, **(d)** CNN (CT) loss, **(e)** ResNet18 (MRI) loss, and **(f)** ResNet18 (CT) loss.

Figure 2 presents the training and validation loss curves for all experimental configurations, including CNN, ResNet18, and multimodel fusion across the combined dataset. For CNN and ResNet18 trained on CT scans, the loss decreased smoothly over epochs, stabilizing at 0.12–0.15, with validation curves closely tracking the training curves, indicating minimal overfitting. MRI-based models demonstrated even stronger convergence, with validation losses consistently below 0.10 after the initial epochs, reflecting MRI features' higher discriminative power for tumor detection. The multimodel fusion further enhanced learning stability, with both CNN and ResNet18 fusion models converging rapidly and achieving the lowest final loss values across experiments. These patterns confirm that the proposed framework not only achieves high classification accuracy but also maintains robust and stable optimization dynamics across diverse modalities.

**Figure 3:**
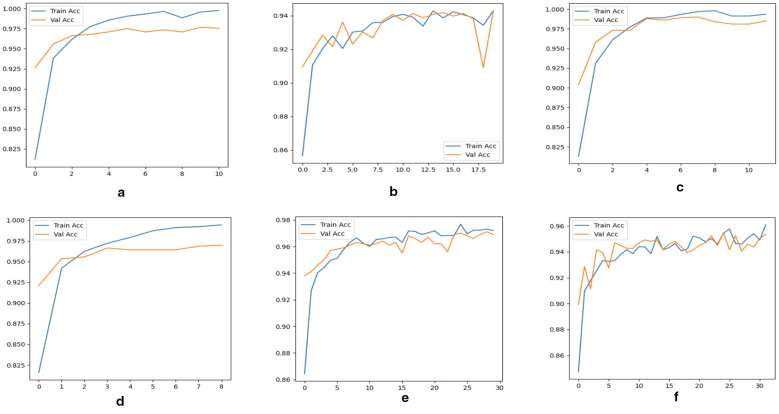
Training and validation accuracy curves for CNN, ResNet18, and multimodel fusion models across combined datasets. The *x*-axis represents training epochs, and the *y*-axis represents classification accuracy. The curves demonstrate consistent convergence behavior, minimal overfitting, and stable generalization across all configurations. **(a)** Multimodel CNN accuracy, **(b)** multimodel ResNet18 accuracy, **(c)** CNN (MRI) accuracy, **(d)** CNN (CT) accuracy, **(e)** ResNet18 (MRI) accuracy, and **(f)** ResNet18 (CT) accuracy.

Figure 3 illustrates the training and validation accuracy curves for CNN, ResNet18, and multimodel fusion models. For CT datasets, CNN and ResNet18 achieved stable convergence, with final validation accuracies of 94%–95%, indicating reliable but slightly lower performance compared with MRI. MRI-based models demonstrated superior performance, with validation accuracies consistently above 97% for both CNN and ResNet-18, confirming MRI's advantage in capturing tumor-specific structural features. The multimodel fusion achieved the best results, with validation accuracy approaching 99%, showing minimal gaps between training and validation sets and indicating strong generalization and minimal overfitting. These accuracy curves corroborate the classification metrics, confirming that multimodal integration and hybrid architectures substantially enhance robustness and clinical applicability for brain tumor classification.

Table 2 presents the confusion matrices for CNN and ResNet18 models across multimodal datasets. In the multimodal CNN, the model correctly classified 827 healthy and 985 tumor cases, resulting in 112 misclassifications, for an overall accuracy of 94%. By comparison, the multimodal ResNet18 achieved improved performance, correctly identifying 839 healthy and 1037 tumor samples, reducing errors to 48 cases and achieving 98% accuracy. In single-modality experiments, the MRI-based CNN achieved 99% accuracy, correctly classifying 370 healthy and 619 tumor cases. At the same time, the MRI-based ResNet18 also performed strongly, achieving 97% accuracy with only 30 misclassifications. Similarly, the CT-based CNN achieved 97% accuracy with balanced-class predictions. In contrast, the CT-based ResNet-18 achieved 95% accuracy, with slightly more classification errors than MRI. These results show that CNN generalize better to independent CT and MRI classification, whereas ResNet-18 achieves superior performance in multimodal fusion by leveraging pretrained high-level features.

**Table 2:** Confusion matrix components for CNN and ResNet18 models across different imaging modalities.

Model	Modality	TP	TN	FP	FN
CNN	Multimodal	1037	839	29	19
	MRI	619	370	5	6
	CT	447	446	11	20
ResNet18	Multimodal	985	827	41	71
	MRI	606	364	17	13
	CT	427	453	16	28

**Figure 4:**
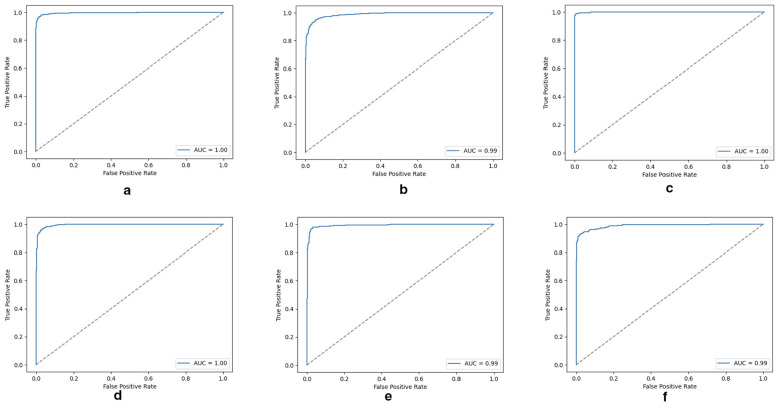
ROC curves with corresponding AUC scores for CNN, ResNet18, and multimodel fusion across the combined dataset. All models achieve near-perfect discrimination ability, with AUC values ranging from 0.99 to 1.00. **(a)** Multimodel CNN ROC, **(b)** multimodel ResNet18 ROC, **(c)** CNN (MRI) ROC, **(d)** CNN (CT) ROC, **(e)** ResNet18 (MRI) ROC, and **(f)** ResNet18 (CT) ROC.

Figure 4 presents the ROC curves for CNN, ResNet-18, and multimodal fusion across the combined datasets. The multimodal CNN (Figure 4a) achieved an AUC of 0.99, while the multimodal ResNet18 (Figure 4b) slightly outperformed with an AUC of 1.00, confirming its robustness when integrating CT and MRI data. For MRI-only experiments, both CNN (Figure 4c) and ResNet18 (Figure 4e) achieved excellent separability with AUCs of 0.99 and 0.99, respectively, reflecting the high tissue contrast of MRI. In CT-only settings, CNN (Figure 4d) and ResNet18 (Figure 4f) maintained strong performance with AUC values of 1.00 and 0.99. Overall, all models achieved near-perfect classification ability, with multimodal ResNet18 consistently showing the best generalization, highlighting the advantage of combining pretrained features with multimodal data.

The comparative analysis in Table 3 highlights the performance of the proposed models against recent state-of-the-art approaches for brain tumor classification. It is important to note that differences in datasets, class distributions, preprocessing pipelines, and evaluation protocols inherently limit direct quantitative comparisons across studies. Therefore, the comparison presented in Table 3 is intended to provide a contextual and qualitative benchmark rather than a strictly equivalent evaluation. Prior studies, such as Gundogan (2025) and Rahman et al. (2025), employed hybrid CNN architectures enhanced with explainable AI techniques, achieving accuracies of 98.90–99.77% on MRI datasets. Similarly, Kar and Singh (2025) introduced MBTC-Net, a multimodal CNN with attention mechanisms, reporting 99.34% accuracy on CT–MRI inputs, while Usha et al. (2024) and Aamir et al. (2025) demonstrated strong performance using multimodal and ensemble frameworks. Several recent studies report very high accuracy values; however, these results are often obtained under controlled conditions, such as single-modality MRI data, curated datasets, or specific preprocessing strategies, which may not fully reflect real-world clinical variability. In contrast, the proposed framework achieves competitive performance under a more heterogeneous and practically relevant setting. The CNN trained from scratch achieves 99.00% accuracy on MRI data and 96.97% on CT data, while the multimodal fusion framework achieves 97.66% accuracy on a combined CT–MRI dataset. Unlike many prior works that focus on single-modality inputs, our approach explicitly evaluates performance across both CT and MRI modalities with varying characteristics, noise levels, and acquisition protocols. Achieving comparable performance under these conditions demonstrates improved robustness and generalization capability. Furthermore, the effectiveness of the proposed method is attributed to the complementary nature of the architectures: the CNN captures modality-specific, low-level tumor features, particularly effective for MRI, while ResNet18 contributes high-level pretrained representations that enhance learning across heterogeneous inputs. Consequently, the fusion model provides a more stable and clinically adaptable solution, maintaining consistent performance across varying imaging modalities. This highlights the practical advantage of the proposed framework for real-world deployment, where multimodal and variable-quality inputs are common.

**Table 3:** Comparison of the proposed method with recent brain tumor classification approaches.

Study	Method/model	Modality	Dataset	Accuracy	Remarks
Gundogan (2025)	Hybrid CNN + XAI	MRI	Private + Kaggle	99.77%	Single-modality, curated dataset
Rahman et al. (2025)	Deep CNN + SHAP	MRI	Clinical MRI	98.90%	MRI-only, controlled setting
Kar and Singh (2025)	MBTC-Net	CT + MRI	Public multimodal dataset	99.34%	Attention-based multimodal model
Usha et al. (2024)	TumNet (multimodal CNN)	CT + MRI	Private dataset	99.94%	Dataset-specific optimization
Aamir et al. (2025)	Ensemble CNN framework	MRI	Kaggle MRI datasets	98.00%	MRI-only evaluation
Rasa et al. (2024)	Fine-tuned transfer learning models	MRI	Public MRI	99.96%	Controlled experimental setup
Albalawi et al. (2024)	Customized CNN	MRI	Clinical MRI	99.00%	MRI-focused pipeline
Lu et al. (2025)	CNN + segmentation framework	MRI	Non-contrast MRI	98.30%	Single-modality setting
**Proposed**	CNN (from scratch)	MRI	Multimodal dataset	**99.00%**	Domain-adaptive learning
**Proposed**	CNN (from scratch)	CT	Multimodal dataset	96.97%	Challenging CT modality
**Proposed**	CNN + ResNet18 Fusion	CT + MRI	Combined dataset	**97.66%**	Robust multimodal setting
**Proposed**	Fusion (ResNet pipeline)	CT + MRI	Combined dataset	94.28%	Transfer learning baseline

Note that results are not directly comparable due to differences in datasets and evaluation protocols. The best results are shown in bold.

## 5 Discussion and analysis

The experimental evaluation highlights the strong potential of deep learning models for brain tumor classification across CT, MRI, and multimodal datasets, achieving consistently high performance across all metrics. Among the single-modality models, CNNs trained on MRI scans achieved the best results, with 99% accuracy and precision, and recall values close to 0.99, confirming MRI's superior sensitivity for soft-tissue visualization compared to CT, where the best performance reached 97%. In CT-based experiments, both CNN and ResNet demonstrated reliable performance but exhibited slightly higher misclassification rates, consistent with the lower contrast and higher noise levels inherent in CT imaging. Interestingly, when integrating modalities, the multimodal CNN achieved a solid 94% accuracy. In contrast, the multimodal ResNet18 significantly outperformed it with 98% accuracy and an AUC of 1.00, reflecting the advantage of transfer learning in extracting robust high-level features across heterogeneous inputs. Confusion matrices further reinforced these findings: the multimodal ResNet-18 produced only 48 misclassifications, compared with 112 for the multimodal CNN. At the same time, MRI-only CNN results were almost flawless, with minimal errors in distinguishing between healthy and tumor cases. Loss and accuracy curves demonstrated smooth convergence across all experiments, with no major signs of overfitting. In contrast, ROC curves showed near-perfect discriminative ability across all configurations, with AUC values ranging from 0.99 to 1.00. These results suggest that CNNs excel at single-modality tasks, particularly MRI, whereas ResNet-18 is better suited for multimodal fusion, as its pretrained backbone generalizes across imaging domains. Clinically, the implications are clear: MRI-based AI systems provide highly accurate and reliable tumor detection, but multimodal fusion, particularly with ResNet18, offers a balanced pathway toward robust clinical deployment by leveraging the complementary strengths of CT and MRI. Nonetheless, the slight drop in CT-based performance underscores the importance of continued work on modality harmonization and domain adaptation to fully unlock the potential of multimodal medical imaging for automated diagnostics.

## 6 Conclusion

This study presented a comprehensive evaluation of deep learning models for brain tumor classification using CT, MRI, and multimodal imaging datasets. By systematically comparing custom CNNs trained from scratch with pretrained ResNet18 architectures and their multimodal fusion, we demonstrated that both the model architecture and the choice of modality critically influence performance. The results revealed that CNNs achieved exceptional accuracy in single-modality tasks, particularly on MRI data, reaching 99% classification accuracy. CT-based models also achieved strong results, with accuracies up to 97%, though performance was slightly lower due to inherent limitations of CT imaging in soft-tissue characterization. Importantly, multimodal fusion highlighted the advantage of integrating complementary information. While the multimodal CNN achieved 94% accuracy, the multimodal ResNet18 attained 98% with an AUC of 1.00, underscoring the benefit of leveraging pretrained feature extractors for heterogeneous inputs. The overall findings suggest that CNNs are highly effective on homogeneous, single-modality datasets, whereas transfer learning with ResNet18 provides superior generalization in multimodal settings. These results have direct clinical implications, emphasizing that MRI-based AI tools are particularly suitable for precise tumor detection. At the same time, multimodal systems offer enhanced robustness and reliability for deployment in diverse clinical environments. Future work will focus on improving CT performance through advanced preprocessing and domain adaptation, and on extending multimodal frameworks with explainable AI to further bridge the gap between deep learning models and clinical' interpretability.

## Data Availability

Publicly available datasets were analyzed in this study. This data can be found at: https://www.kaggle.com/datasets/murtozalikhon/brain-tumor-multimodal-image-ct-and-mri.
